# Transformation of *Anaplasma phagocytophilum*

**DOI:** 10.1186/1472-6750-6-42

**Published:** 2006-10-31

**Authors:** Roderick F Felsheim, Michael J Herron, Curtis M Nelson, Nicole Y Burkhardt, Anthony F Barbet, Timothy J Kurtti, Ulrike G Munderloh

**Affiliations:** 1 Department of Entomology, University of Minnesota, St. Paul, MN, 55108, USA; 2 Department of Pathobiology, College of Veterinary Medicine, University of Florida, Gainesville, FL, 32611, USA

## Abstract

**Background:**

Tick-borne pathogens cause emerging zoonoses, and include fastidious organisms such as *Anaplasma phagocytophilum*. Because of their obligate intracellular nature, methods for mutagenesis and transformation have not been available.

**Results:**

To facilitate genetic manipulation, we transformed *A. phagocytophilum *(*Ap*) to express a green fluorescent protein (GFP) with the Himar1 transposase system and selection with the clinically irrelevant antibiotic spectinomycin.

**Conclusion:**

These transformed bacteria (GFP/Ap) grow at normal rates and are brightly fluorescent in human, monkey, and tick cell culture. Molecular characterization of the GFP/Ap genomic DNA confirmed transposition and the flanking genomic insertion locations were sequenced. Three mice inoculated with GFP/Ap by intraperitoneal injection became infected as demonstrated by the appearance of morulae in a peripheral blood neutrophil and re-isolation of the bacteria in culture.

## Background

*Anaplasma phagocytophilum *(*Ap*, formerly the Human Granulocytic Ehrlichiosis agent) is a common tick borne obligate intracellular pathogen with an uncommon tropism for host granulocytes. While much has been made of the physiologic stability of the intracellular environment, vector transmission requires extraordinary flexibility to bind and infect the variety of cell types encountered in the travels of the pathogen within and between vector and host(s). Remarkably, *Ap *and the related rickettsial pathogens accomplish this feat with small genomes.

Tracking tissue distribution, cellular binding, entry, and intracellular development of these organisms would be greatly augmented by expression of fluorescent proteins, but genetic transformation of obligate intracellular bacteria has only been accomplished in a few cases [1-5]. Obstacles to transformation of obligate intracellular pathogens include: DNA delivery while retaining viability of extracellular bacteria, efficient reintroduction of the transformed bacterial population into host cells, selection (given the limited number of antibiotics ethically applicable to a pathogen), and the limited efficiency of homologous recombination and transposition systems. Recent development of the mariner transposase Himar1, which can function in many organisms [6-14] has effectively diminished this last obstacle. The further development of hyperactive Himar1 mutants, as detailed by Lampe et.al. [15], has made this transposition system capable of driving insertional mutagenesis systems [8,9,11-13,16-19].

Here we describe the first successful transformation of *Anaplasma phagocytophilum*. We used Himar1 transposition to produce *Ap *transformants that express green fluorescent reporter protein GFPuv at levels useful for imaging. These GFPuv expressing *Ap *(GFP/Ap) transformants grow readily in a variety of cell types and in a manner so far indistinguishable from the growth of the non-transformed parental strain. Also like the parental strain, they are infectious for laboratory mice.

## Results

### Southern Blot, confirmation PCR and rescue cloning

To detect the presence of the GFPuv – Spec^r ^DNA in the fluorescent bacteria, a set of PCR primers not used in the plasmid construction (Forward UV-SS confirmation PCR, Reverse UV-SS confirmation PCR) was used to amplify a 700 bp product which spans the junction between the two coding sequences. Using total DNA isolated from GFP/Ap infected HL-60 as the template, the primers readily detected the presence of the inserted DNA in 25 cycles. Samples without primers or without template gave no signal (data not shown).

**Figure 1:**
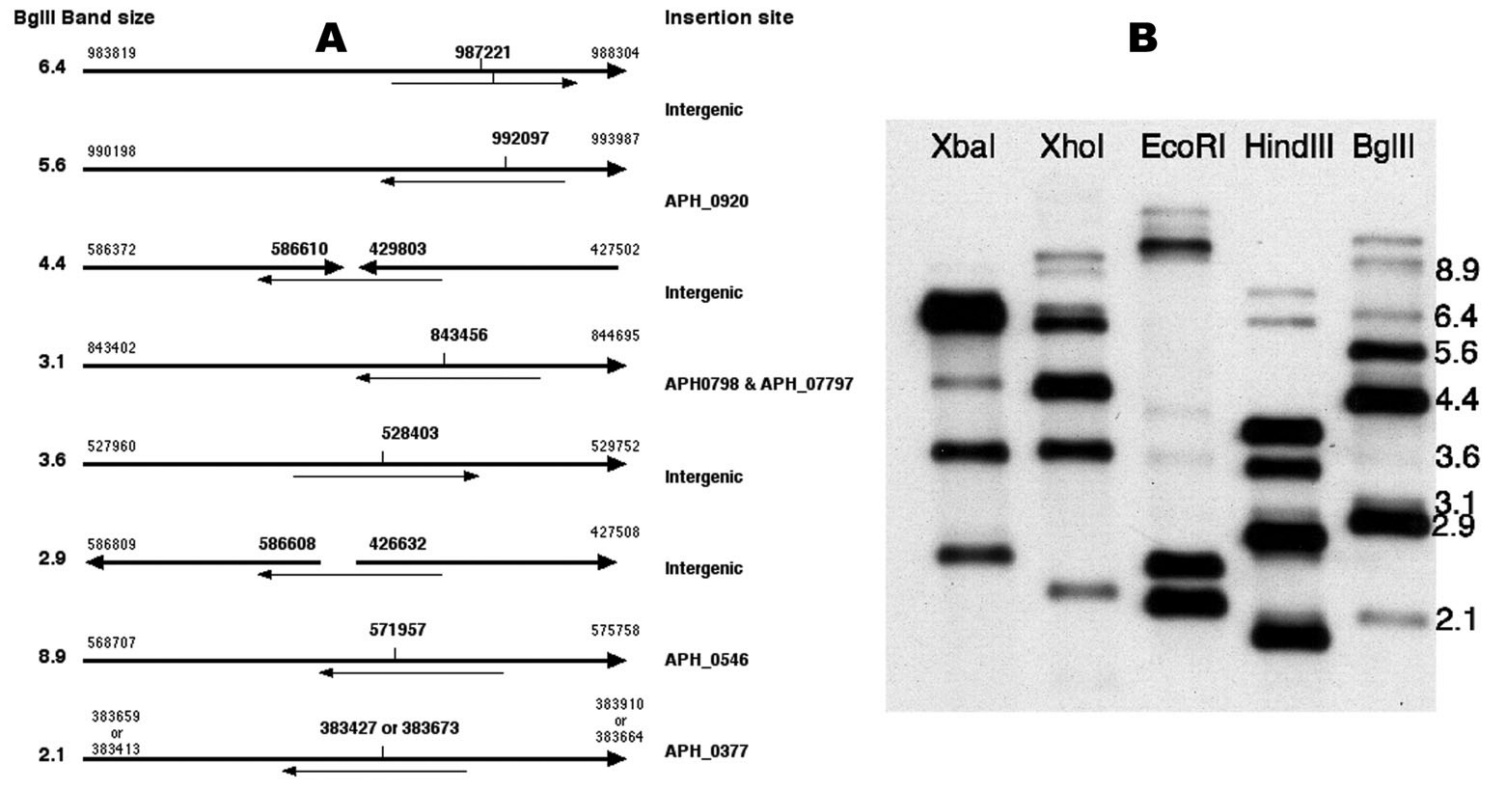
Transposition insertion diagram and Southern blot. (A) This diagram represents eight *Ap *genomic locations and direction of the transposon (arrow under the line) as determined by rescue cloning from GFP/Ap genomic DNA and sequence mapping onto the Ap HZ genome. Four insertions were found in putative coding sequences and four were in intergenic sites. The band sizes on the left of the diagram correspond to bands in the BglII digest lane of the Southern blot. (B) Southern blot of restriction digested GFP/Ap genomic DNA probed with a GFPuv coding sequence probe. The labels indicate restriction enzyme used and the numbers on the right indicate the sizes of visible bands in the BglII lane.

Transposon insertion points map to the following positions on the *Ap *HZ genome[20]: 992097, 987221, 843456, 528403, 571958 (Figure 1A). Two examples were found in which one end of the transposon maps to a different location than the other end; 586610 or 429803, and 586608 or 426632 (Figure 1A). We attribute this to possible recombination following transposition or differences between the HGE1 and HZ *Ap *strains. Another insertion maps to a repeat region of a virB6 family member gene and could either be inserted at 383427 or 383673. Limited sequence data prevents discrimination between the two.

Southern analysis of restriction digested genomic GFP/Ap DNA using a GFPuv probe detected 7–9 bands of varying intensities in most of the digests (Figure 1B). The pattern of bands in the BglII digest and their intensities is consistent with the sizes and numbers of BglII fragments obtained in the rescue cloning from genomic DNA isolated from GFP/Ap. The bands detected from the BglII digest are approximately 2.1, 2.9, 3.1, 3.6, 4.4, 5.6, 6.4, 8.9 and 12 kb in size. The numbers of rescue clones from each size class are 2.1 kb – 1 clone, 2.9 kb – 6 clones, 3.1 kb – 6 clones, 3.6 kb – 1 clone, 4.4 kb – 20 clones, 5.6 kb – 5 clones, 6.4 kb – 2 clones, 8.9 kb – 1 clone. The Southern blot shows the 4.4 kb band to be the most intense, and most rescue clones recovered were of this class. Insertion events for each BglII fragment size class were sequenced out from both ends of the transposon into flanking genomic DNA, and into genomic DNA from the vector used in the rescue cloning. All insertions had the expected TA dinucleotides at the junctions of transposon repeats and genomic DNA sequence. For sequence comparison, we used the recently sequenced *Ap *HZ strain to map the sequences obtained from the rescued clones onto the *Ap *genome.

The transformation efficiency at this point is three to thirty transformants per electroporation of bacteria isolated from one T-75 flask of infected HL-60 cells.

### *In vitro *growth and imaging

**Figure 2:**
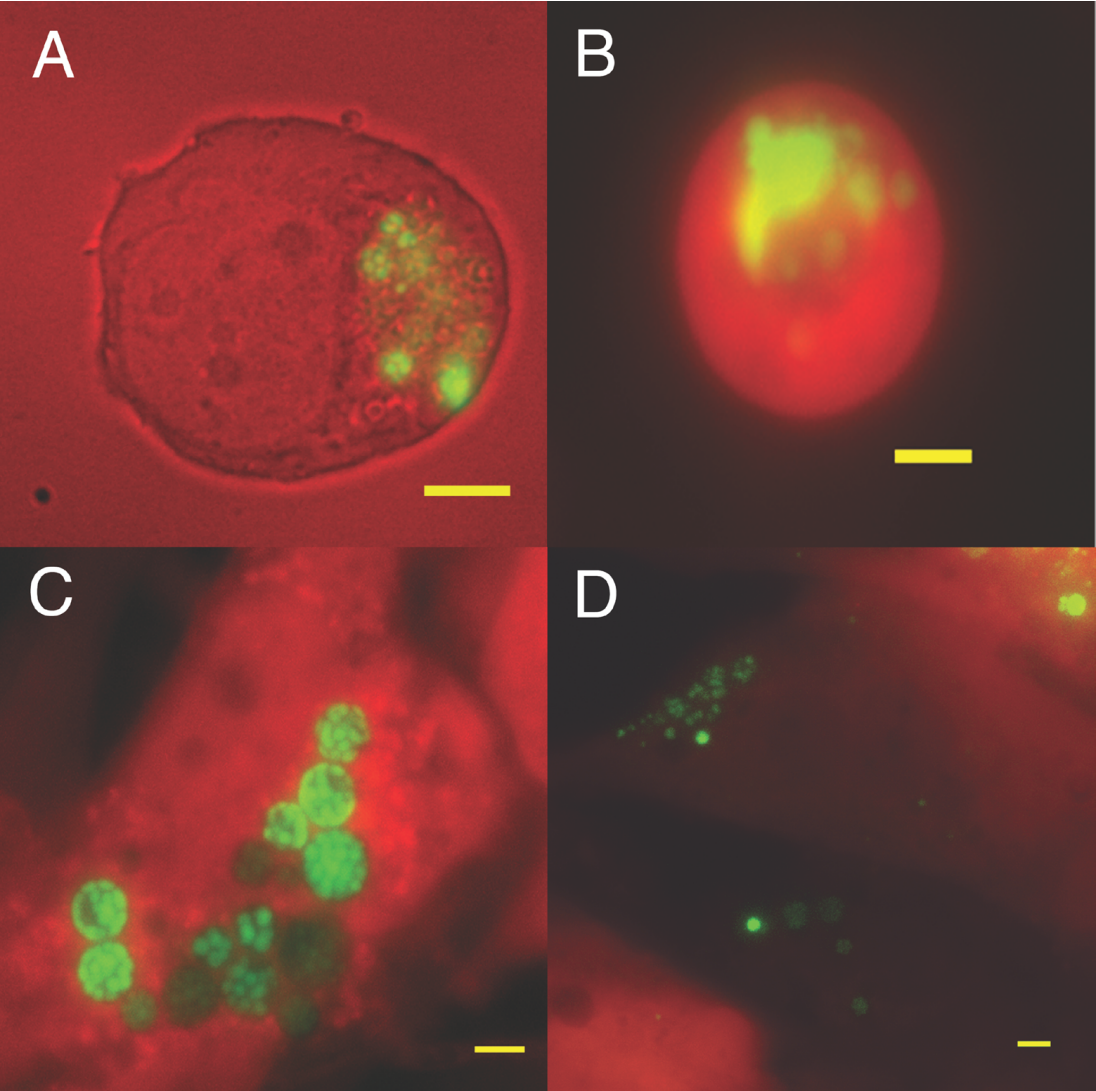
Images of GFP/Ap growing within various host cells. HL-60 (A) with partial bright field to illuminate the non fluorescent host cell. Tick cell ISE6 (B) expressing DSred and containing indistinct bacteria in morulae. Monkey RF/6A (C) and human HMEC-1 (D) endothelial cells expressing mCherry and containing GFP/Ap morulae with distinct bacteria. Bars 5 μm.

Approximately 10^5 ^of GFP/Ap infected HL-60 (Figure 2A) were inoculated into mCherry/RF6A, mCherry/HMEC-1, and DSred/ISE6 growing in 35 mm glass bottom culture dishes (MatTek) and incubated as detailed above. After 48 hours, endothelial cells were rinsed to remove HL-60 cells and imaged. Imaging revealed fully developed morulae with a variety of fluorescence intensities and characteristic, pleomorphic *Ap *morphology (Figure 2C and 2D). The visualization of live endothelial and HL-60 cell cultures revealed morulae with dramatically symmetrical arrangements of bacteria. DsRed2/ISE6 cells imaged after 27 days showed bacterial inclusions as characteristically indistinct masses. (Figure 2B)

### Infection of mice

Three mice – one C3H SCID and two immunocompetent (C57BL/6) – challenged with GFP/Ap, became infected. Following ip inoculation with GFP/Ap infected HL-60, a characteristic *Ap *inclusion was seen in a neutrophil of the C3H scid mouse, and GFP/Ap was cultured from the peripheral blood of all three mice.

## Discussion

Transformation of *Ap *represents an important step in the development of methods for the genetic manipulation of human and animal anaplasmosis agents. The inability to employ many molecular techniques in the study of these emerging infectious agents has hampered the normally rapid progression of research. The availability of hyperactive Himar1 transposases and the methods described herein should allow the routine transformation of *Ap *and related organisms and accelerate work in this area.

Successful bacterial transformations require a mechanism of genomic remodeling with a high enough efficiency to be effective with a reasonable population of bacteria and a means to select rare transformants. Transformation of a pathogen should not involve the use of clinically relevant antibiotics or constructs that are likely to allow horizontal transfer of resistance to other organisms. Spectinomycin resistance and the Himar1 transposon system fulfill both of these requirements. The major use of spectinomycin is presently one of 21 antimicrobial drugs used for treatment of gonococcal infections [21]. We could find no reports of spectinomycin use in anaplasmosis. *Ap *contains no known plasmids or mobile elements that might enable resistance transfer. The "cut and paste" mechanism of mariner type transposase plasmid systems such as Himar1, in which the transposase sequence is not incorporated into the target genome, are not conducive to horizontal transfer. The two-plasmid system employed in these transformations may provide an additional element of safety by reducing the likelihood of accidental genomic transposase integrations.

Our choice of a promoter to control expression of the transposase and GFP was driven by the analysis of the tr promoter using quantitative PCR [22]. The tr promoter is one of the few characterized in *Anaplasma *and we have demonstrated it to be expressed in bacteria grown in both mammalian and tick cells. The upstream out-of-frame start codon located between the start of transcription and the start of translation was removed to increase expression of GFP and spectinomycin resistance. Presumably it is present in wild type *Am *to attenuate the level of tr protein produced. The efficiency of Himar1 transposition is a function of transposon size, with a 38% decrease for every 1-kb increase in transposon size [23]. To keep the transposon under 2 kb the GFPuv reporter and spectinomycin resistance genes were driven by a single tr promoter via translational coupling. Future studies should allow the exploration of promoters that are regulated by environmental changes.

Insertion site cloning and sequencing reveals that the transposon was inserted in intergenic regions four times and interrupted real or putative coding sequences four times. The transposition event at position 992097 is located 45 base pairs upstream of the stop codon of an ankyrin repeat protein gene (APH_0928). As a result the last 14 amino acids have been changed from wild type but the protein is otherwise unaffected. The transposition event at position 843456 lies inside two small overlapping putative open reading frames (APH_0798 and APH_0797). The transposition event at position 571957 disrupts a putative open reading frame (APH_0546). Lastly, the transposition event at position 383427 or 383673 lies in (APH_0377) a VirB6 family member. All four of these insertions into putative coding sequences appear to be tolerated in *Ap *cultured in HL-60 cells.

Regarding the stability of transformants; The bacteria remain fluorescent green and spectinomycin resistant after more than 40 passages in HL-60 cells (with or without spectinomycin selection). They are a population of transformants at this point (i.e. not clonal) so we expect the proportion of individual insertions relative to one another may change over time.

The development of reporter genes such as green fluorescent protein has greatly accelerated the study of changing biological systems, both *in vitro *and *in vivo*. *Ap *that constitutively express GFPuv can be used for *in vitro *studies of *Ap *binding, entry, morula development, and cell/cell transfer using standard widefield epifluorescence and confocal techniques for live cell observation and imaging. In previous work using histochemistry and immunostaining we have demonstrated that the development of *Ap *in tick cells differs strikingly from its growth in human cells [24]. In human cells, *Ap *forms morulae containing bacteria that are individually visible. In tick cells the morulae often become enlarged and ill defined [25]. Imaging of GFP/Ap in ISE6 tick cells reveals the same characteristics (Figure 2B). GFP/Ap grown in HL60 and endothelial cells (Figure 2A,C,D) display the compact, well-defined morulae and pleomorphism seen in non-transformed *Ap *[24].

To date, the visual study of *Ap in vitro *and *in vivo *has relied upon static fixed samples stained by standard histological techniques. Such studies of static specimens can never give a complete picture of the dynamic processes of bacterial growth and development within host cells or animals. Indeed, many aspects of morula development and *Ap*/host-cell interaction can only be studied by continuous observation over time, made possible with fluorescent reporter proteins. The combination of fluorescent *Ap *and host cells, each expressing a contrasting fluorescent reporter protein, will allow observation of the development of live *Ap *into morulae, and the passage of *Ap *from cell to cell in an adherent cellular system that is especially amenable to microscopic imaging. Towards these ends, the GFP expression obtained in these transformants is bright, and is useful for live cell imaging. The number of distinctly differentially bright *Ap*, when compared to the number of insertions sequenced, suggests that the site of transposon insertion influences expression, as has been found in other systems.

A central goal in our efforts to establish a method for transforming *Ap *has been to produce fluorescent bacteria. Live, fluorescent bacteria can be readily imaged in cultured host cells, and *in vivo *within the cells of the ticks and mammals *Ap *naturally infects. It has been our experience that when passed extensively *in vitro *(approximately > 15 passes) *Ap *loses its infectivity for animals (unpublished data). Because our initial efforts at transforming *Ap *have required a substantial amount of *in vitro *culture (e.g. to generate sufficient quantities of bacteria for transformation experiments and to cultivate potential transformants), we have been concerned that the transformants that arise will be poorly infective for animals. This has not been the case, however. In preliminary experiments we have found that these transformed *Ap *behave like untransformed parental bacteria; they invade and grow within tick (ISE6) and primate (RF/6A, HMEC1, HL-60) cells and infect mice.

## Conclusion

In this study, we have described a simple method for transformation and selection of *Ap*. The resulting transformants grow normally in all *in vitro *systems in common use for the culture of these organisms and have successfully infected laboratory mice; suggesting behavior similar to the parental strain. The GFP transformants will prove useful for observation of bacterial binding, entry, growth and cellular exit. These transformation methods should allow gene knock out by random mutagenesis, and the method of spectinomycin selection may prove useful for specific gene knockout by homologous recombination.

## Methods

### Cell and Bacterial culture

The human promyelocytic leukemia cell line HL-60 (American Type Culture Collection, Manasssas, VA, USA; ATCC CCL-240) was used to propagate *Ap *strain HGE1 [26]. HL-60 cells, infected and uninfected, were maintained in RPMI1640 (Bio-Whittaker, Walkersville, MD, USA) with 10% heat-inactivated fetal bovine serum (FBS, Harlan, Indianapolis, IN, USA) and 25 mM HEPES in 5% CO_2 _in humidified air at 37°C.

Additional mammalian cells employed in this study were: endothelial lines RF/6A (ATCC CRL-1780), from the retina choroid endothelium of a normal fetal rhesus monkey (*Macaca mulatta*), and the human microvascular endothelial cell line HMEC-1 [27]. All cells were maintained as specified above for HL-60 cells. Adherent cells were detached using trypsin (Gibco, Grand Island, NY, USA), and diluted five-fold once a week.

The Tick cell line ISE6, isolated from embryos of the black-legged tick, *I. scapularis*, was grown in L15B300 with 5% tryptose phosphate broth (Difco Laboratories, Detroit, MI, USA), 5% heat-inactivated FBS (Harlan), and 0.1% bovine lipoprotein concentrate (MP Biomedical, Irvine, CA, USA), pH 7.2. Medium for infected cultures was additionally supplemented with 25 mM HEPES and 0.25% NaHCO_3_, and the pH adjusted to 7.5–7.7. ISE6 cultures were maintained at 34°C. *Ap *were subcultured by transferring 1/50th of an infected culture to a new flask containing sterile host cells. [25].

### Host cell Transformation

RF/6A, HMEC-1 and ISE6 cell lines were transformed to express mCherry [28] or DsRed2 (Clontech, Mountain View, CA), under the control of the chicken beta-actin promoter and flanked by the transposase recognition sequences, using the Sleeping Beauty Transposon system [29]. DNA was delivered into sub confluent monolayers using Effectene (Qiagen, Valencia, CA) according to the manufacturers instructions. After several days, selection with G418 sulfate was begun and continued until cells not expressing fluorescent protein were absent for two weeks.

The plasmid used to impart fluorescence to host cells was constructed by moving the GFP expression cassette from pVITRO4-NEO-GFP/LacZ (Invivogen, San Diego, CA) as a NheI – NotI DNA fragment, into pT-HB (a gift from P. B. Hackett) between the Sleeping Beauty IR/DR sequences. The GFP expression cassette contains the CAG promoter driving GFP followed by an FMDV IRES, the EM7 promoter and the neomycin resistance gene. An *E. coli *origin of replication is also on the DNA fragment. To obtain red fluorescent host cells, the coding sequence for GFP was replaced by those of mCherry or DsRed-2.

### Plasmid construction

All enzymes were obtained from New England Biolabs (Beverly, MA), Promega (Madison, WI), or Stratagene (La Jolla, CA), unless stated otherwise. PCR was performed using PfuUltra HS (Stratagene). Electrophoresis and blot transfer buffers were prepared as described previously [30], unless stated otherwise. All primers (Table 1) were from MWG Biotech (High Point, NC) or Integrated DNA Technologies (Coralville, IA). Standard molecular techniques were used throughout [31].

#### Transposase expression plasmid

**Figure 3:**
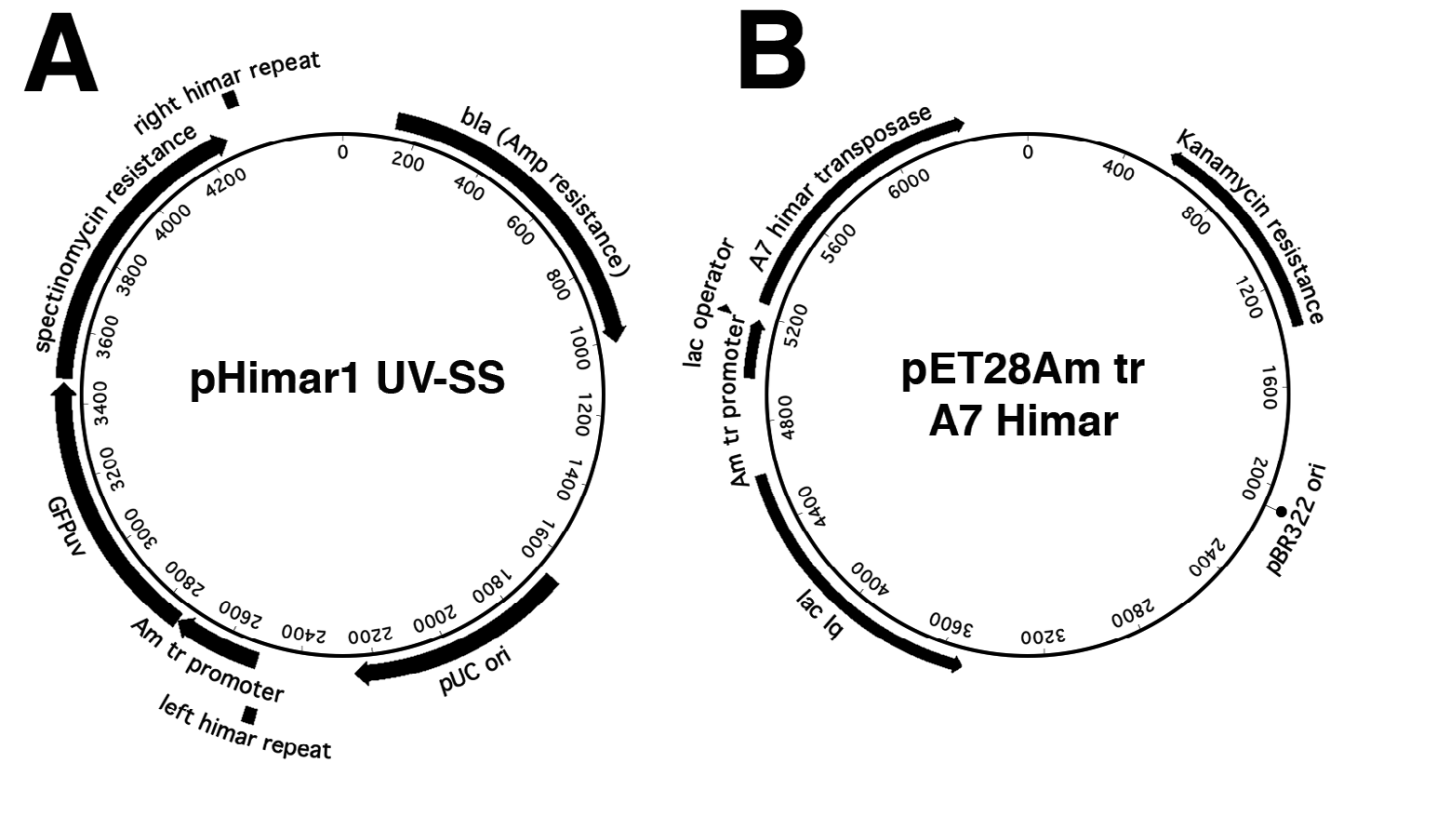
Physical maps of the Himar1 transposon and transposase plasmids. (A) pHIMAR1-UV-SS carries the *A. marginale *promoter tr driving expression of GFPuv and spectinomycin resistance between the left and right Himar transposon repeats. (B) pET28AMTR-A7-HIMAR contains the A7 hyperactive mutant of the Himar1 transposase also driven by the Am tr promoter.

The vector used to express the Himar1 transposase was pET28 (Novagen, Madison, WI) due to the presence of the *lacIq *gene and *lac *operator sequence, to minimize expression of the transposase in *E. coli*. The T7 promoter of pET28 was replaced with the tr promoter from *Anaplasma marginale *(*Am*) [22] by PCR of the vector using the primers pET28 T7 replace PCR and pET28 lacO PCR, and PCR of the promoter using the primers 5' Amtr pro Himar1 and 3' Amtr pro Himar1. Both PCR products were cut with BglII and ligated, creating pET28AMTR. The Himar1 transposase coding sequence was moved into this vector as a NcoI-HindIII fragment from pBADA7 [15] to create pET28AMTR-A7-HIMAR (Figure 3B).

#### Transposon plasmid

The promoter chosen to drive expression of GFPuv was the tr promoter from *Am*. This promoter was isolated from *Am *genomic DNA as an EcoRI – BamHI fragment using PCR and primers 5' Am tr pro and 3' Am tr pro. Base number 19 in the 3' *Am *tr promoter primer was substituted with a T to remove the upstream out of frame start codon. The DNA fragment was cut with EcoRI and BamHI and cloned into the same restriction sites of pMOD-2 (Epicentre, Madison WI). The BamHI site was removed from the coding sequence of GFPuv in pGFPUV (Clontech) using the QuickChange method (Stratagene). The GFPuv coding sequence was isolated from the modified pGFPUV using PCR and the primers 5' GFPuv PCR and 3' GFPuv PCR phos. The spectinomycin resistance coding sequence was isolated from a derivative of pMON9443 [32] using PCR and the primers 5' S-S PCR phos and 3' S-S Xba PCR. The GFPuv PCR product was cut with BamHI, the Spec PCR product was cut with XbaI and both were ligated into the pMOD-2 with *Am *tr promoter vector cut with BamHI and XbaI. To increase expression of GFPuv, the BamHI site upstream of the ATG was replaced with AT rich sequence by site directed mutagenesis, using the QuickChange method (Stratagene) and primers Bam re SDM A and Bam re SDM B. The expression cassette *Am *tr GFPuv-Spec was moved from the pMOD based plasmid into pLITMUS-HIMAR1-REPEATS, as detailed below, using EcoRI and HindIII.

To generate pLITMUS-HIMAR1-REPEATS, the restriction sites between and including EcoRI and KpnI were removed from pLITMUS28 (New England Biolabs) by restriction digestion and blunting with Pfu DNA polymerase. The Himar1 left and right repeat oligonucleotide sets were annealed and cut with either BglII or XhoI. Himar1 repeat elements were ligated into the modified pLITMUS28 using the BglII and XhoI sites. The DNA from this ligation was cut with EcoRI and HindIII and the expression cassette was moved from the pMOD based plasmid described above into this pLITMUS-HIMAR1-REPEATS plasmid as an EcoRI – HindIII fragment, creating pHIMAR1-UV-SS (Figure 3A).

Prior to electroporation into *Ap*, the plasmid DNAs were grown in the *dam/dcm *mutant *E. coli *strain GM2163 (New England Biolabs), isolated using endofree Maxi-prep kits (Qiagen), and methylated with *Ap *protein extracts as in [33].

### Bacterial transformation and selection

Cell density of *Ap *infected HL-60 in upright flasks was maintained between 1–5 × 10^5^/ml by 20 to 60-fold dilution of fully (>90% of cells) infected cultures with uninfected cells. Culture infection was monitored by microscopic examination of Giemsa stained slides (Cytospin, Shandon, Sewickley, PA) prepared from small samples of the cultures. When it was determined that greater than 90% of cells were infected, host cell free *Ap *were prepared by needle aspiration, 2.0 μm glass fiber filtration (Whatman), and twice washed in 270 mM sucrose. The pelleted bacteria were placed on ice and resuspended in a small volume of 270 mM sucrose. One μg of pET28-AMTR-A7-HIMAR and 1 μg of pHIMAR1-UV-SS (described above) were added to the resuspended bacteria and a 50-μl aliquot was pulsed once (4 to 5 ms, 1.2 kV, 400 Ohms, 25 μF) with a Gene Pulser II (Bio-Rad, Hercules, Calif.) in a 0.1-cm-gap electroporation cuvette. Electroporated bacteria were immediately combined with 5 million HL-60 in 5 ml of medium and incubated overnight at 37°C. The next day spectinomycin (100 μg/ml) selection began. When bacteria exhibiting spectinomycin resistance were evident, cultures were monitored for green fluorescence by observing wet mounts on an Olympus BH2-RFCA microscope with epifluorescent illumination and a fluorescein isothiocyanate filter set. Images were collected with a CFW-1310M CCD (Scion, Frederick, Maryland) and ImageJ (Rasband, W.S., ImageJ, National Institutes of Health, Bethesda, Maryland, USA) with the VisiCapture plugin from Scion. HL-60 contained green fluorescent morulae with individual bacteria distinctly visible (Figure 2A).

### PCR and Southern blot detection of transposons in *Anaplasma *genomic DNA

PCR was performed to confirm the GFPuv – Spec^r ^expression cassette was within GFP/Ap DNA using AmpliTaq Gold DNA polymerase (Roche, Indianapolis, IN), and primers Forward UV-SS confirmation PCR and Reverse UV-SS confirmation PCR. DNA was isolated from GFP/Ap-infected HL-60 cultures using the AquaPure DNA isolation kit (Bio-Rad). Cycling conditions were as follows: 94°C for 5 min; 94°C for 30 sec, 52°C for 30 sec, and 74°C for 45 sec for 25 cycles, followed by a final extension at 74°C for 5 min. Amplicons were electrophoresed on a 0.8% agarose gel and stained with ethidium bromide. For Southern blots, 100 ng of GFP/Ap DNA was digested with XbaI, XhoI, EcoRI, HindIII, BglII, or EcoRV, electrophoresed on a 1% agarose gel, and transferred overnight onto Zeta Probe GT genomic membrane (Bio-Rad) in 0.4 M NaOH. The blots were rinsed in 3× SSC buffer, baked at 80°C for 30 min, prehybridized at 65°C for 2 hours in 2× block buffer [34], and hybridized overnight at 65°C with GFPuv digoxigenin-labeled probes prepared with the PCR DIG Probe Synthesis kit (Roche) and end-terminal primers. Blots were washed twice in 2× SSC-0.1% SDS for 5 min at 22°C, once in 0.5× SSC-0.1% SDS for 15 min at 65°C, once in 0.25× SSC-0.1% SDS for 15 min at 65°C, and once in 0.1× SSC-0.1% SDS for 15 min at 65°C. They were then developed with the DIG Wash and Block Buffer Set and CDP-Star detection reagent according to the protocol of the manufacturer (Roche), and exposed to Kodak X-OMAT AR film.

### Cloning and sequencing of transposon integration sites

Genomic GFP/Ap DNA was isolated from purified bacteria using the AquaPure DNA isolation kit (Bio-Rad), cut with BglII and ligated into pLITMUS28 cut with BglII (BglII lies outside the transposon in pHIMAR1-UV-SS). *E. coli *was electroporated with this ligation and 46 colonies were picked from SOB plates containing ampicillin 75 μg/ml, spectinomycin 50 μg/ml and streptomycin 50 μg/ml. Plasmid DNA, isolated from cultures grown from the colonies, was cut with BglII and electrophoresed on agarose gels to size the inserts. Plasmid DNAs from each insert size class were sequenced with the vector primers M13 FOR and M13 REV and primers that bind inside the transposon and face outward (UV-SS up and out, UV-SS down and out) to allow sequencing of the transposon-genomic DNA junction. The insertions were mapped using the published genomic sequence of the *Ap *HZ strain [20] (note that the transformed strain described here is HGE1).

### Microscopy

Microscopic images of GFP/Ap were obtained of cells and bacteria cultured in 35 mm glass bottom culture dishes (MatTek, Ashland, MA). Cells were examined on a TE2000-U Inverted microscope (Nikon, Melville, N.Y.) using epifluorescent illumination, piezo actuated z movement (Mad City Labs, Madison WI), with FITC and TRITC filter sets. Monochrome serial z planes were collected with a Cascade 1 K (Photometric) camera. To clearly image host cells and bacteria, collected images from the red and green emission channels were processed by maximum projection of z planes and adjustment of the look up table using Metamorph (Molecular Devices).

### GFP/Ap infection of mice

A C3H scid mouse was challenged by intraperitoneal (ip) injection with 2 × 10^5 ^GFP/Ap infected HL-60 cells suspended in 500 μL cell culture medium. Six days later the mouse was humanly sacrificed, blood was aseptically drawn by cardiac puncture, and 200 μL inoculated into a culture of HL-60 cells. A blood smear was also prepared and Giemsa stained. Microscopic examination revealed an *Ap *infected neutrophil. After eight days incubation, cytocentrifuged cells from the blood-inoculated HL-60 culture were shown by Giemsa stain to be *Ap *infected. Five days later, when most cells were infected, a wet mount was prepared (~2 × 10^6 ^cells in 15 μL medium overlaid with a cover slip) and microscopically examined by epifluorescence. Green fluorescent *Ap *bacteria with normal morula morphology – that commonly seen with wild-type *Ap *– were clearly seen in cells.

Two inmmunocompetent (C57BL/6) mice were then inoculated ip with the scid mouse-isolated GFP/Ap. Twenty-four hours later, one mouse was sacrificed, blood collected by cardiac puncture, a smear prepared, and an HL-60 culture inoculated. On day 12 the second mouse was sacrificed and blood was drawn for culture and microscopy analysis. Both blood samples cultured in HL-60 yielded GFP/Ap. No infected cells were found in either blood smear.

## Authors' contributions

RFF designed and developed expression strategies and constructs, carried out all DNA manipulations, and assisted with drafting the manuscript. MJH designed study approaches and methods, performed the cell culture, electroporations, microscopy, and drafted the manuscript. CMN carried out the mouse work and participated in manuscript preparation. NYB did the Southern blot and participated in manuscript preparation. TJK and UGM performed the analysis of transformed anaplasma in tick cell culture. AFB contributed intellectually and by provision of DNA constructs to the initiation of the study. UGM conceived and coordinated the study, participated in experimental design, and assisted with drafting the manuscript. All authors have read and approved the final manuscript.

## Figures and Tables

**Table 1:** Primer List

pET28 T7 replace PCR	CCTATAGTGAGTCGTATTAATTTCGCGGGATCG
pET28 lacO PCR	phos-GGAATTGTGAGCGGATAACAATTCCCCTC
5' Amtr pro Himar1	GTTGTTAGATCTGGCTCCTCCTAGAACGATCGC
3' Amtr pro Himar1	phos-GATAACTTAGGATAAATCATACATAATGTTAATGCAACAG
5' Am tr pro	GTTGTTGAATTCGGCTCCTCCTAGAACGATCGC
3' Am tr pro	GTTGTTGGATCCTATCCCTTATGTTACTCAGATAACTTAGGATAAATCA
GFPuv SDM A	CCACAACATTGAAGATGGTTCCGTTCAACTAGCAGACC
GFPuv SDM B	GGTCTGCTAGTTGAACGGAACCATCTTCAATGTTGTGG
5' GFPuv PCR	GTTGTTGGATCCATGAGTAAAGGAGAAGAACTTTTCACTG
3' GFPuv PCR phos	phos-AATCCCTATTTGTAGAGCTCATCCATGCCATGTGTAATC
3' S-S Xba PCR	GTTGTTTCTAGATTATTTGCCGACTACCTTGGTGATCTCGCCTTTC
5' S-S PCR phos	phos-AAATTATGAGGGAAGCGGTGATCGCCGAAGTATCGACTC
Bam re SDM A	CTGAGTAACATAAGGGATATTATAATGAGTAAAGGAGAAGAAC
Bam re SDM B	GTTCTTCTCCTTTACTCATTATAATATCCCTTATGTTACTCAG
Himar1 right repeatA	TGTTGAAGCTTAGACCGGGGACTTATCAGCCAACCTGTTACTCGAGTTGT
Himar1 right repeatB	ACAACTCGAGTAACAGGTTGGCTGATAAGTCCCCGGTCTAAGCTTCAACA
Himar1 left repeatA	TGTTGAGATCTAACAGGTTGGCTGATAAGTCCCCGGTCTGAATTCTTGTT
Himar1 left repeatB	AACAAGAATTCAGACCGGGGACTTATCAGCCAACCTGTTAGATCTCAACA
UV-SS up and out	CATCACCTTCACCCTCTCCACTGAC
UV-SS down and out	CAGCCCGTCATACTTGAAGCTAGGC
Forward UV-SS confirmation PCR	AAAGATGACGGGAACTACAAGACG
Reverse UV-SS confirmation PCR	CACAACAATGGTGACTTCTACAGCG
